# Regulatory T cells specifically suppress conventional CD8αβ T cells in intestinal tumors of APC^Min/+^ mice

**DOI:** 10.1007/s00262-020-02540-9

**Published:** 2020-03-17

**Authors:** Louis Szeponik, Paulina Akeus, William Rodin, Sukanya Raghavan, Marianne Quiding-Järbrink

**Affiliations:** Department of Microbiology and Immunology, Institute of Biomedicine, Sahlgrenska Academy, University of Gothenburg, Gothenburg, Sweden

**Keywords:** Regulatory T cells, APC^min/+^, Colon cancer, Tumor-infiltrating lymphocytes, Anti-tumor immunity

## Abstract

The presence of activated T cells in colorectal cancer tissues is a strong predictor of patient survival. Our previous studies have shown that regulatory T cells (Treg) are able to reduce T cell transendothelial migration in vitro and accumulation of effector T cells in intestinal tumors in vivo in the murine APC^Min/+^ model for microsatellite stable intestinal tumors. In this study, we investigated the effect of Treg depletion on the density and effector functions of different TCRαβ^+^ and TCRγδ^+^ T cell populations in intestinal tumors. We used the APC^Min/+^\DEREG mouse model, which harbor a diphtheria toxin receptor under the control of the FOXP3 promoter, to deplete Treg in tumor bearing mice. We found that the density of conventional TCRαβ^+^CD8αβ^+^ T cells was significantly increased in Treg-depleted tumors in comparison with Treg-proficient tumors. Furthermore, TCRαβ^+^CD8αβ^+^ T cells showed increased proliferation and activation as well as increased Granzyme B and IFN-γ production in Treg-depleted tumors. In sharp contrast, the densities and effector functions of TCRαβ^+^CD8αα^+^ T cells and TCRγδ^+^ T cells remained unchanged by Treg depletion. We also documented a distinct population of IL-17A^+^TNF^+^ TCRγδ^+^CD8^−^ T cells in tumors, which were not affected by Treg depletion. We conclude that Treg depletion affects only conventional TCRαβ^+^CD8αβ^+^ T cells in intestinal tumors, while unconventional T cells and T cells in unaffected tissue are not altered. Immunotherapies aimed at depleting Treg from tumors may thus be a viable option for reinvigoration of conventional cytotoxic T cells with a Th1 cytokine profile.

**Electronic supplementary material:**

The online version of this article (10.1007/s00262-020-02540-9) contains supplementary material, which is available to authorized users.

## Introduction

Colorectal cancer (CRC) is one of the most common cancer diseases worldwide [1]. Overall, there has been an improvement in the survival of CRC patients over the last decades [2]; nevertheless, while some patients respond well to therapies, others do not. Microsatellite instability (MSI), resulting from mutations leading to inactivation of the mismatch repair genes, is used as a diagnostic marker in the clinic. The 10–15% of patients that are MSI-High (MSI-H) usually show a better disease-free and overall survival [3] than the remaining 85–90% of microsatellite stable (MSS) CRCs. MSS tumors typically show chromosomal instabilities and mutations in the adenomatous polyposis coli (APC) gene giving rise to both familial adenomatous polyposis and most of the sporadic cancers [4].

It is now well known that the immune system plays an important part in tumor initiation and progression and that immune cells can be both anti- and protumorigenic [5]. MSS tumors are usually characterized by poor infiltration of Th1-type lymphocytes, which are classically defined as CD4^+^ T cells secreting IFN-γ [6]. Th1 cells promote cytotoxicity by CD8^+^ T cells, which in turn can also secrete substantial amounts of cytokines. In contrast, MSI-H tumors generally show a high infiltration of Th1 type lymphocytes [7, 8], probably caused by the generation of neo-antigens through frame-shift mutations [9]. The Th1 response is beneficial for the prolonged survival and outcome of CRC patients [10] as are tumor-specific cytotoxic T cells [11], which are important mediators of tumor regression. Furthermore, unconventional TCRγδ T cells can have anti-tumor effects by production of type 1 cytokines and cytotoxic potential [12, 13]. The continuous antigenic stimulation in the tumor microenvironment can also favor exhaustion of cytotoxic T cells and thereby reduce the killing of tumor cells [14]. Exhaustion is a state of T cell non-responsiveness and results from a complex network of different cell types, which express inhibitory receptors and secrete cytokines that eventually lead to suppression of immune effector functions. Overcoming the exhaustion of cytotoxic T cells by immunotherapy targeting checkpoint inhibitors has shown success in melanoma, lung cancer and MSI CRC [15, 16]. On the other hand, not much has improved for patients with MSS CRC who do not respond to the new immune therapies. New alternative therapies and combination therapies are needed to generate a response in these patients.

In addition to exhaustion, regulatory T cells (Treg) in the tumor microenvironment can reduce anti-tumor immunity. In CRC, Treg accumulation can lead to suppression of protective immune responses [17] and inhibition of migration of lymphocytes [18]. There have been different views on whether Treg in CRC are beneficial or detrimental as there are a number of studies showing either positive or negative correlation between infiltration of FoxP3^+^ cells and patient outcome [19–21]. However, a notable study by Saito et al. [22] shows that Treg with high and low FOXP3 expression have different suppressive ability. The study establishes a negative effect of Treg infiltration on patient outcome, and this might explain the discrepancies in other studies. Nevertheless, the effect of tumor-infiltrating Treg on different cytotoxic and unconventional T cell subsets still remains incompletely characterized.

In this study, we used the APC^Min/+^ mouse model for intestinal tumors, which can be considered as a model of early MSS CRC [23]. Even though the APC^Min/+^ mice have defects in immune regulation, most notably splenomegaly and thymic regression, there is no intestinal inflammation or major alterations in intestinal immune function compared to wild-type mice [24, 25]. As in human tumors, CD8^+^ T cell infiltration is important for control of tumor growth in the APC^Min/+^ mice [26], while Treg infiltration and IL-17 signaling promotes tumorigenesis [27, 28]. There is a profound accumulation of Treg in the tumors of APC^Min/+^ mice [25], as in human CRC, and we have also shown that Treg suppressive function is intact in the APC^Min/+^ mice [29]. To examine the effect of Treg depletion on tumor immunity, APC^Min/+^ mice were crossbred with DEREG (**DE**pletion of **REG**ulatory T cell) mice. In our previous work, we have shown that depletion of Treg leads to migration and accumulation of CXCR3^+^ CD8^+^ T cells in the tumors [29]. We now show that Treg depletion leads to increased activation and effector molecule production only in conventional TCRαβ^+^CD8αβ^+^ T cells, while unconventional T cell subsets are unaffected.

## Materials and methods

### Mouse strains and breeding

APC^Min/+^ mice on a C57BL/6 background and DEREG mice [30] were bred to generate APC^Min/+^/DEREG mice and APC^Min/+^ mice at the Department of Experimental Biomedicine, University of Gothenburg. Four weeks after birth APC^Min/+^ genotype was confirmed by PCR and DEREG (Foxp3-GFP) phenotype by flow cytometry as previously described [29, 30]. Animals were kept under specific pathogen-free conditions in filter top cages, and all procedures were approved by the regional animal ethics committee in Gothenburg.

### In vivo Treg depletion and anti-PD-1 antibody injection

Treg in both female and male 18-week-old APC^Min/+^/DEREG mice were depleted by i.p. injections of 0.5 μg diphtheria toxin (DT) on day 1, 2, 8, and 9 as previously described [29]. As controls, APC^Min/+^ mice were identically treated. DT treatment depleted at least 90% of Treg in blood, intestine and tumors by day 3 and 10 but did not reduce tumor burden [29]. In the present study, there was actually an increase in tumor burden in Treg-depleted mice (supplementary Fig. 1a). Mice were killed and organs harvested on day 12 post-start of DT treatment. In some mice, PD-1 or isotype antibodies (kindly provided by Dr. Rene de Waal Malefyt, Merck Inc.) were injected i.p together with DT on day 1 and 9, and alone on day 5 with a dose of 5 mg/kg per mouse. Combined treatment with DT and PD-1 antibody or solely PD-1 antibody had no effect on tumor burden (supplementary Fig. 1b).

### Lymphocyte isolation

Intraepithelial and lamina propria (LP) lymphocytes from small intestinal tumors and unaffected intestine were isolated as follows. Briefly, Peyer’s patches were removed and tumors were cut out; the remaining tumor-free small intestine was cut into small pieces (3–5 mm). Epithelial cells were removed by HBSS medium containing EDTA (5 mM, Invitrogen), equine serum (10%, Hyclone GE Healthcare), and HEPES (15 mM, Invitrogen) for 3 × 15 min at 37°. This fraction also contained intraepithelial lymphocytes (IEL), which were used for further analysis. The remaining tissue was digested with collagenase D (0.03 U/mL, Roche) in RPMI-1640 (Gibco) media supplemented with equine serum (20%) and HEPES (15 mM, Gibco) for 1 h. Subsequently, the released cells were recovered and the remaining tissue was mixed twice in the same media for 1 min with gentleMACS™ Dissociator (Miltenyi). Dissociated tissue was filtered through a 70-µm nylon mesh to recover remaining lymphocytes and reduce debris content.

### Lymphocyte stimulation

Lymphocytes from unaffected tissue and tumor tissue were incubated with PMA (50 ng/mL, Sigma-Aldrich), ionomycin (1 µg/mL, Sigma-Aldrich), Brefeldin A (1 µg/mL, BD), and Monesin (1,95 µM, BD) in IMDM (+ 25 mM HEPES, Gibco) with 10% FCS (Hyclone GE Healthcare), 0.1 mg/mL Gentamycin (BioWhittaker), 2 mM l-Glutamine (Merck), 50 µm 2-Mercapthoethanol (Gibco) for 5 h at 37 °C and 5% CO_2_. Unstimulated cells from unaffected lamina propria served as controls. For degranulation assays, CD107a-BV421 (1D4B, BD) was added to the stimulation culture from the beginning in a 1:200 dilution.

### Flow cytometry

Single cell suspensions were stained with Live/dead Aqua (Molecular Probes, Inc.). Antibodies were titrated in pilot experiments on small intestinal lymphocytes and splenocytes to find optimal dilutions achieving good signal separation and low background signal, and are listed in supplementary Table 1. Surface stainings were performed in Brilliant Stain Buffer (BD). To detect transcription factors, fixation and permeabilization were performed with the Foxp3/Transcription Factor Staining Buffer Set (eBioscience). For cytokine detection, the Intracellular Fixation and Permeabilization Buffer Set (eBioscience) was used. Cells were acquired with BD LSRFORTESSA X-20 and analyzed in FlowJo v10 (BD). Gates were set using FMO controls as a guidance. Additionally, unstimulated cells were used to set the gates for CD107a and intracellular cytokines. Supplementary Fig. 2a shows the initial gating for CD45^+^ live lymphocyte. Supplementary Fig. 2b shows FMO and unstimulated controls for CD107a and Granzyme B, and supplementary Fig. 2c shows FMO controls for PD-1 and Tim-3. SPICE (NIH) was used for the analysis of combinations of cytokines and activation/checkpoint molecules.

### Immunohistochemistry

OCT-embedded small intestinal tissue with tumors was cut in 7-µm-thick sections and stored at − 20 °C. Sections were fixed with 2% formaldehyde in PBS for 10 min. Endogenous peroxidase activity was blocked with NaN_3_ (PeroxAbolish, Biocare Medical) for 1 h. Avidin/Biotin blocking (Biolegend) was performed for 10 min each. Subsequently, tissue was incubated with CD8β-FITC (clone 53-5.8, Biolegend) and TCRγδ-biotin (ebioGL3, ebioscience) for 1 h, followed by incubation with anti-FITC-HRP (Thermofisher) antibody for 40 min. The CD8β signal was developed with Tyramide-AF488 (Thermofisher) in 1X Plus Amplification buffer (Perkin Elmer) for 4 min. The HRP activity was blocked with NaN_3_ for 30 min. Sections were then incubated with Streptavidin-HRP (Thermofisher) for 15 min, and the TCRγδ signal was developed with Tyramide-AF594 (Thermofisher) as above. The sections were incubated with CD8α-BV480 (53-6.7, BD) for 1 h. The tissue was stained with DAPI (Invitrogen) and mounted in ProLong Antifade glass (Invitrogen). All incubations were performed at room temperature, and antibodies were diluted in PBS with 0.1% FCS. Tissue sections were scanned with the Metafer Slide Scanning Platform (Axio Imager.Z2 Microscope and 20×/0.8/air objective, Zeiss). Cells were quantified in Strataquest (TissueGnostics GmbH). We quantified cell subsets in whole tissue scans in the entire tumor area of each section and then normalized to cell numbers per mm^2^.

### Statistical analysis

Statistical significance was evaluated using the Mann–Whitney test for unpaired and Wilcoxon signed-rank test for paired analysis. *p* values of < 0.05 were considered significant. Horizontal lines/bars in the figures show the median. Statistical analyses were performed in GraphPad PRISM software version 8.0 (GraphPad Software).

## Results

### Reduced numbers of CD8αα and CD8αβ T cells in intestinal tumors of APC^Min/+^ mice

**Fig. 1:**
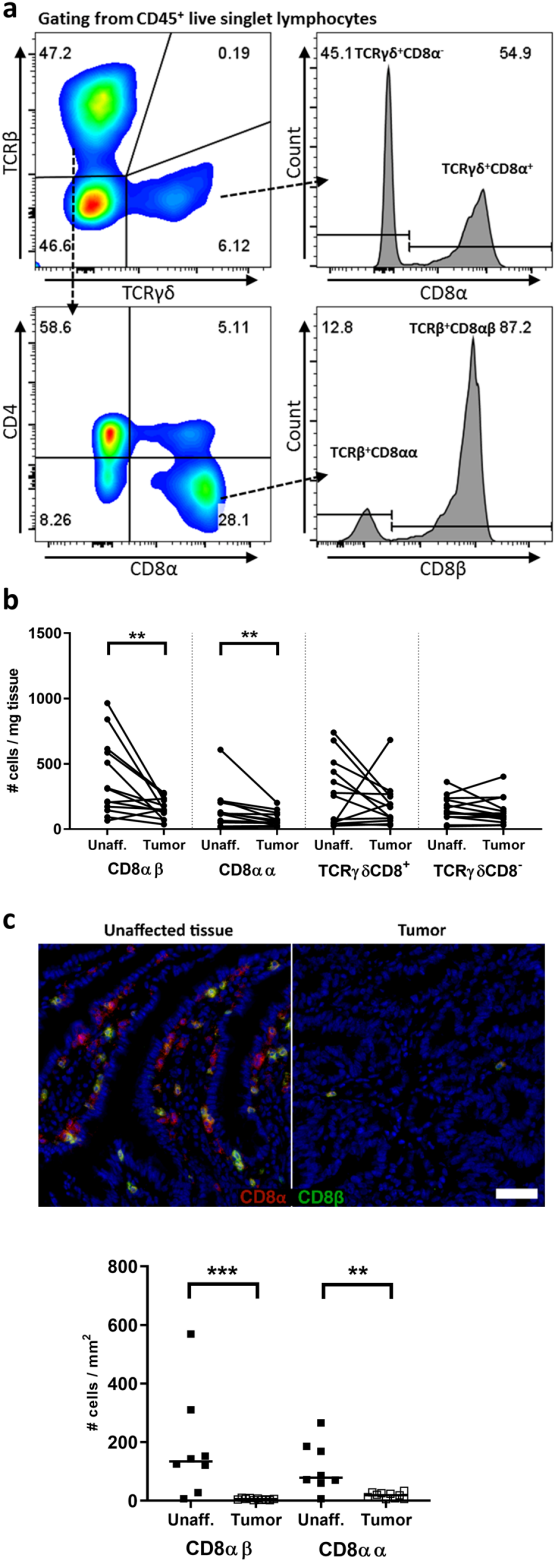
T cell subsets in intestinal tumors and unaffected tissue. Single cell suspensions were isolated from tumor and small intestinal tissue of APC^Min/+^ mice and analyzed for their expression of phenotypic markers by flow cytometry. **a** Flow cytometry gating strategy to distinguish four cell populations: TCRβ^+^CD8αα^+^, TCRβ^+^CD8αβ^+^, TCRγδ^+^CD8^+^, and TCRγδ^+^CD8^−^ T cells. Representative dot plots from a tumor sample. **b** Paired analysis of cell densities of different cell populations in unaffected tissue and tumor tissue of the same mice. **c** Representative immunohistochemistry image of CD8αα and CD8αβ T cells in frozen unaffected tissue and tumor tissue of APC^Min/+^ mice. CD8α in red, CD8β in green, and nuclei in blue, 50-µm scale bar; Lower panel shows quantification of TCRγδ-negative CD8αβ and CD8αα T cells in frozen unaffected tissue and tumor tissue. Symbols represent individual value and lines the median. **p < 0.01, ***p < 0.001 using the Wilcoxon signed-rank test (**a**) and Mann–Whitney test (**c**)

We used APC^Min/+^ mice as a model of early MSS colon cancer and first determined the frequencies and densities of different T cell subsets with cytotoxic potential by flow cytometry in unaffected intestinal tissue and intestinal tumors (see Fig. 1a for gating strategy). These analyses distinguished four major T cell subsets in the tumors: TCRβ^+^CD8αβ^+^ (from now on referred to as CD8αβ), TCRβ^+^CD8αα^+^ (from now on referred to as CD8αα), TCRγδ^+^CD8^+^, and TCRγδ^+^CD8^−^ cells. The frequencies of TCRγδ^+^CD8αβ^+^ and TCRγδ^+^CD4^+^ cells were found to be low (< 1%) from both tumors and unaffected tissue and were hence not investigated further. TCRβ^+^CD8^+^CD4^+^ cells, which only constituted between 0.22 and 3.4% of CD45^+^ lymphocytes in unaffected and 0.2–4% in tumor tissue, were also insufficient for functional experiments. We have previously shown that CD8^+^ T cells are unable to infiltrate the intestinal tumors of APC^Min/+^ mice to any larger extent [25]. Here, we performed a more detailed analysis and show that both subsets of TCRβ^+^CD8^+^ T cells (CD8αα, CD8αβ) are reduced in intestinal tumors from APC^Min/+^ mice compared to unaffected small intestinal tissue when analyzing the number of cells per mg tissue. On the other hand, the numbers of TCRγδ T cells are similar in tumors and unaffected tissue (Fig. 1b). Immunohistochemistry staining confirmed the low infiltration of CD8αα^+^ and CD8αβ^+^ T cells in tumors of APC^Min/+^ mice (Fig. 1c). Interestingly, similar changes in cell density of the different T cell subsets were detected in the IEL fraction when comparing tumors and unaffected tissue (supplementary Fig. 3). In summary, CD8αα and CD8αβ T cells are reduced in the LP and IEL fractions of tumors compared to unaffected small intestinal tissue in the APC^Min/+^ mice.

### The density and activation of CD8αβ T cells is increased in intestinal tumors by Treg depletion

**Fig. 2:**
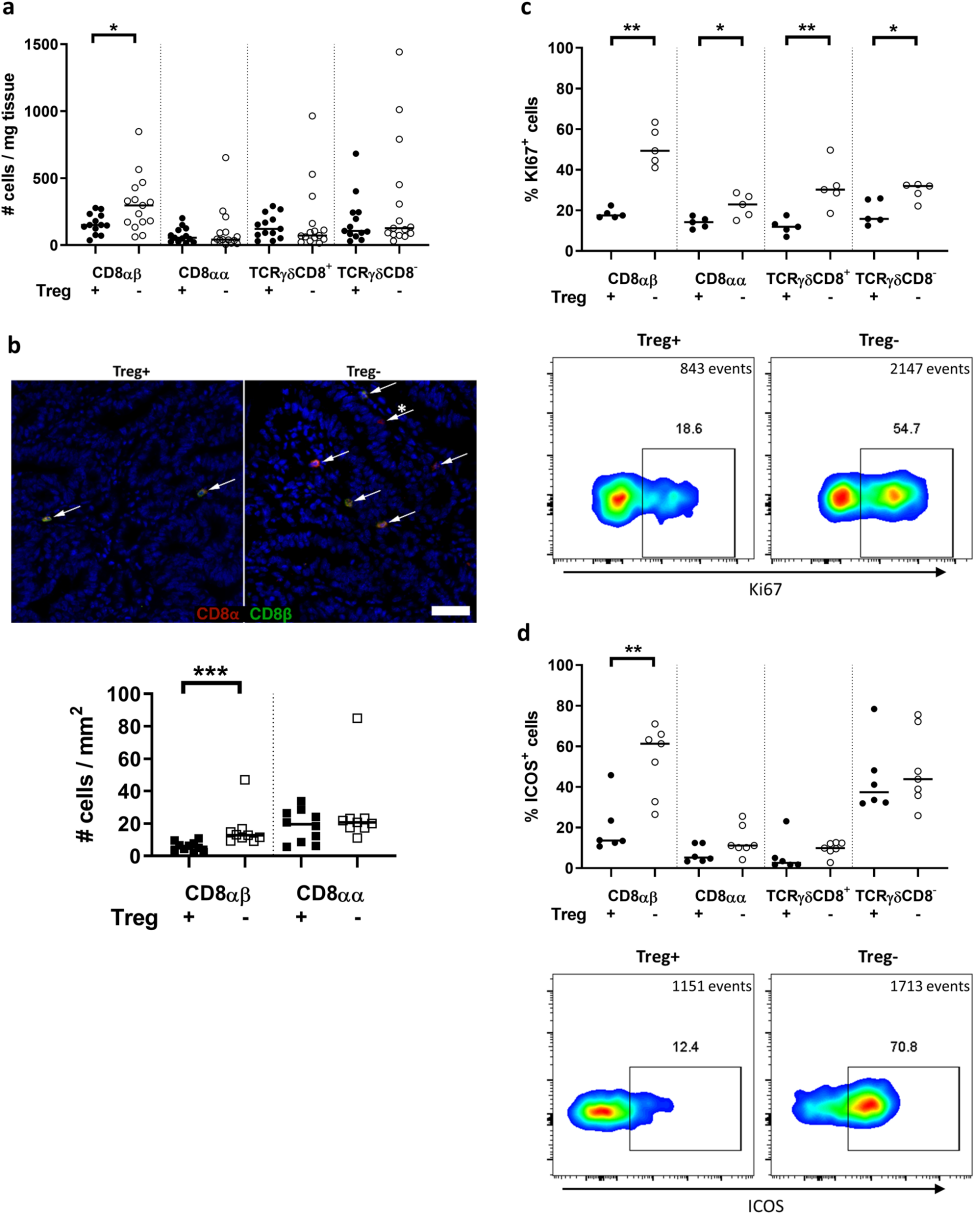
T cell accumulation and proliferation in intestinal tumor tissue following Treg depletion. Single cell suspensions were isolated from tumors of DT-treated APC^Min/+^ and APC^Min/+^/DEREG, and analyzed by flow cytometry. **a** Ex vivo cell densities of different T cell populations in tumor tissue from Treg-proficient (Treg+) and Treg-depleted mice (Treg−). **b** Representative immunohistochemistry image of CD8αα and CD8αβ T cells in frozen tumor tissue of Treg+ and Treg− mice; CD8α in red, CD8β in green, and nuclei in blue; white arrows show CD8αβ T cells, and white arrow with star a CD8αα T cell, 50-µm scale bar. Quantification of TCRγδ**-**negative CD8αβ and CD8αα T cells in frozen tumor tissue from Treg+ and Treg− mice. **c** Upper panel shows ex vivo expression of Ki67 in different T cell populations in tumor tissue from Treg+ and Treg− mice. Lower panel shows a representative dot plot of Ki67 expression in CD8αβ T cells in tumor tissue from Treg+ and Treg− mice. Total events are shown. **d** Upper panel shows ex vivo expression of ICOS in different T cell populations in tumor tissue from Treg+ and Treg− mice. Lower panel shows a representative dot plot of ICOS expression in CD8αβ T cells in tumor tissue from Treg+ and Treg− mice. Symbols represent individual values, and the line represents the median, *p < 0.05, **p < 0.01, ***p < 0.001 using the Mann–Whitney test

Previously, we have demonstrated that short-term depletion of Treg in APC^Min/+^/DEREG mice leads to increased migration of both CD4^+^ and CD8^+^ T cells into intestinal tumors [31], while the cell densities in unaffected tissue remain unchanged, but contribution from the different T cell subsets with cytotoxic potential was not investigated. Thus, we examined the effect of Treg depletion on the density of selected cell subsets in tumors. These assays demonstrated a significant increase of CD8αβ T cells in the Treg-depleted tumors compared to Treg competent tumors, as determined by flow cytometry and immunohistochemistry staining (Fig. 2a, b). In contrast, the density of CD8αα T cells and the two TCRγδ cell populations did not change with Treg depletion. Furthermore, the effect of Treg depletion on T cell accumulation was only evident in the tumors, as there was no change in cell densities in the unaffected small intestinal tissue (supplementary Fig. 4). In the IEL fraction, the cell densities of all the four T cell populations examined also remained unaffected by Treg depletion in both tumors and unaffected tissue (supplementary Fig. 5).

Our previous work showed that accumulation of total CD8^+^ cells in tumors was dependent on both increased migration and increased proliferation of resident cells [31]. We assessed proliferation and activation by the ex vivo expression of Ki67 and ICOS, respectively. We found significantly increased proliferation in all four T cell populations in tumors without Treg (Fig. 2c). However, CD8αβ T cells showed the highest increase in Ki67 expression (2.8-fold). The frequency of ICOS^+^ cells was significantly increased (four-fold), specifically among CD8αβ T cells in the tumor tissue of Treg-depleted mice, compared to Treg-proficient mice (Fig. 2d). A smaller increase in ICOS expression upon Treg depletion was also seen in CD8αβ cells from unaffected tissue (supplementary Fig. 6), while the other T cell subsets did not show any change in ICOS expression.

Taken together, our results show that Treg depletion efficiently increased the proliferation and activation of conventional CD8αβ T cell subset, specifically in tumors, whereas the other T cell populations remained less affected or completely unaffected.

### Treg depletion leads to increased production of Granzyme B in tumor-infiltrating CD8αβ T cells

**Fig. 3:**
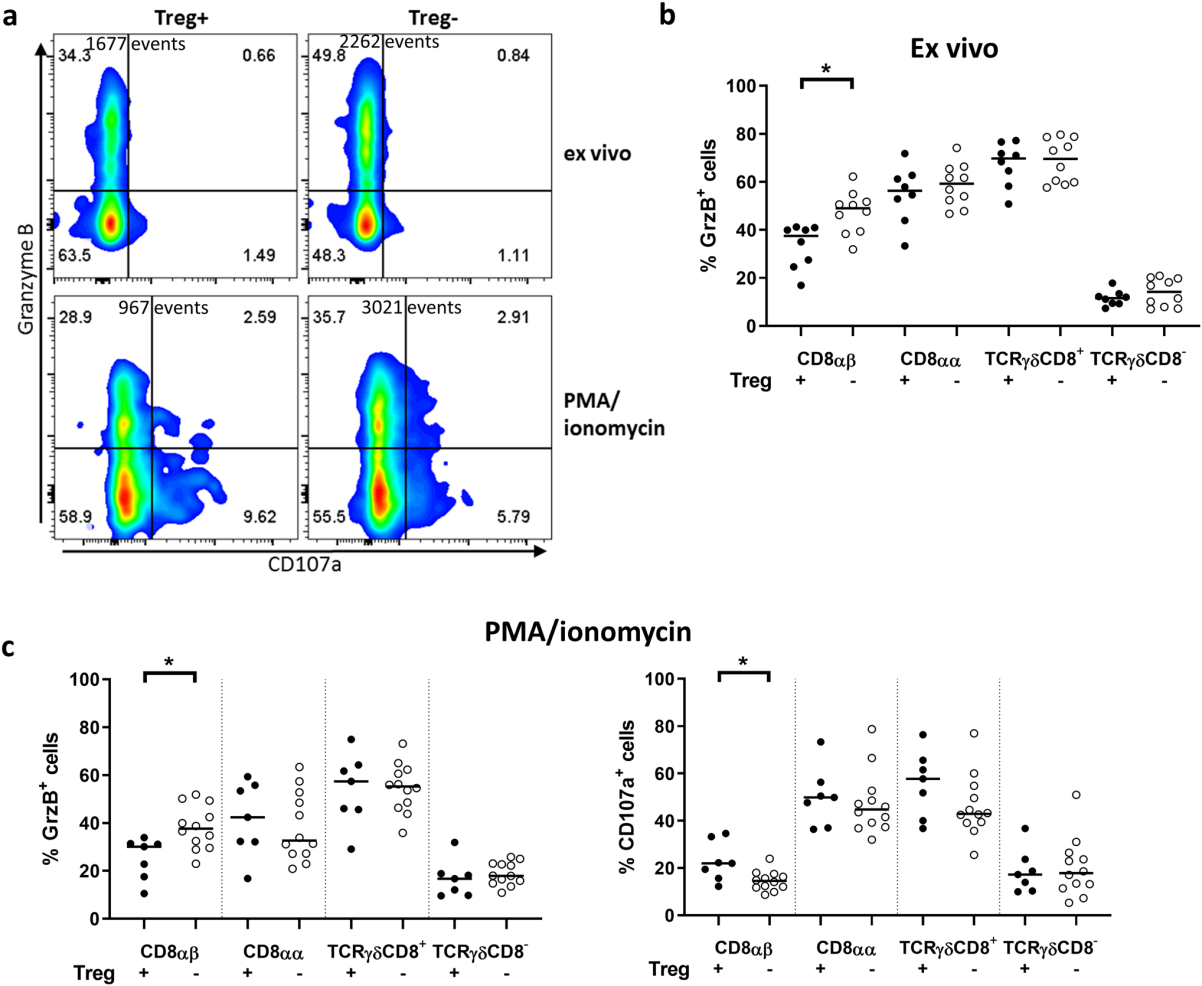
T cell expression of GrzB and CD107a in intestinal tumors following Treg depletion. Single cell suspensions were isolated from intestinal tumors of DT-treated APC^Min/+^ and APC^Min/+^/DEREG mice and analyzed by flow cytometry. **a** Representative flow cytometry plot of CD8αβ T cells expressing GrzB and CD107 ex vivo (upper panel) or after stimulation with PMA/ionomycin (lower panel) in tumor tissue from Treg-proficient (Treg+) and Treg-depleted mice (Treg−). Total events are shown. Frequencies of GrzB-positive cells in different T cell populations in tumor tissue from Treg+ and Treg− mice ex vivo **(b)** and frequencies of GrzB or CD107a positive cells after stimulation with PMA/ionomycin **(c)**. Symbols represent individual values, and the line represents the median, *p < 0.05 using the Mann–Whitney test

To assess the cytotoxic capacity following Treg depletion, intracellular Granzyme B (GrzB) as well as surface expression of CD107a by T cells from unaffected tissue and tumor tissue was investigated (see Fig. 3a). In Treg-proficient mice, there were similar ex vivo frequencies of GrzB expressing cells in the tumors and the unaffected tissues, in both the LP and the IEL fraction, in all the subpopulations analyzed. However, in TCRγδCD8^−^ cells, the expression of GrzB was much lower (~ 10%) than in all other subsets, in both tumors and unaffected tissue (Fig. 3b and supplementary Fig. 7a). Treg depletion significantly increased the frequencies of GrzB^+^ CD8αβ T cells in tumors. (Figure 3b). No other of the examined T cell populations showed a similar increase in GrzB expression. In the IEL fraction, frequencies of GrzB expressing cells were about 20% higher than in the lamina propria, in both tumors and unaffected tissue, but Treg depletion had no effect on the frequency of GrzB^+^ CD8αβ T cells in the IEL fraction (supplementary Fig. 8).

We further assessed the expression of GrzB and CD107a after polyclonal activation of cells with PMA/ionomycin. In vitro activation did not result in any increase of GrzB expression, but Treg depletion still resulted in a significant increase of GrzB^+^ CD8αβ T cells in tumors, but not in any other cell subset or in the unaffected mucosa (Fig. 3c and supplementary Fig. 7b). Finally, the frequencies of CD107a^+^ cells after PMA/ionomycin stimulation were similar in all T cell subsets in tumors after Treg depletion, except for the CD8αβ T cells, that displayed less degranulation in the absence of Treg (Fig. 3c).

In summary, Treg depletion resulted in increased frequency of GrzB^+^ CD8αβ T cells specifically in the tumor, but reduced their degranulation after activation.

### Treg depletion leads to increased production of IFN-γ in tumor-infiltrating CD8αβ T cells

**Fig. 4:**
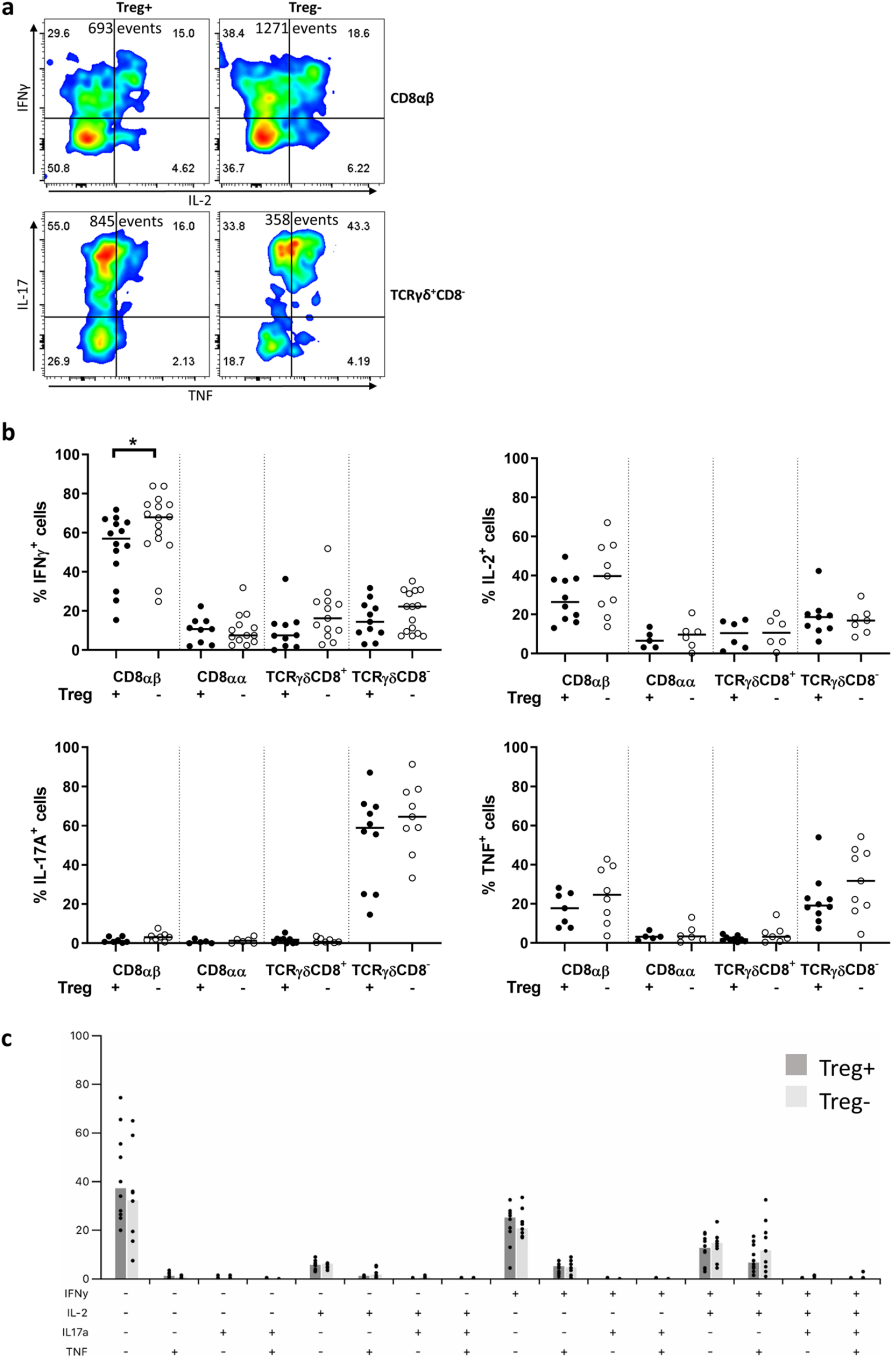
T cell cytokine expression in intestinal tumors following Treg depletion. Single cell suspensions were isolated from intestinal tumors of DT-treated APC^Min/+^ and APC^Min/+^/DEREG mice and analyzed by flow cytometry after PMA/ionomycin stimulation. **a** Representative flow cytometry plot of CD8αβ T cells expressing IFN-γ and IL-2, and TCRγδCD8^−^ T cells expressing IL-17A and TNF in tumor tissue from Treg-proficient (Treg+) and Treg-depleted mice (Treg−). Total events are shown. **b** Frequencies of IFN-γ, IL-2, IL-17A and TNF expressing T cell populations in tumor tissue from Treg+ and Treg− mice. **c** Bar plot of all combinations of the expression of IFN-γ, IL-2, IL-17A, and TNF in CD8αβ T cells from tumor tissue of Treg-proficient (Treg+) and Treg-depleted mice (Treg−). Symbols represent individual values, and the bar represents the median

Next, we assessed the ability of lamina propria T cells from unaffected tissue and tumor tissue to produce some of the cytokines important in tumor immunity (IFN-γ, IL-2, IL-17A, and TNF) (Fig. 4a). The frequencies of IFN-γ^+^ and IL-2^+^ cells among CD8αα, TCRγδCD8^+^ and TCRγδCD8^−^ T cells in tumors were all generally low in comparison with the CD8αβ T cells, where about two-third produced IFN-γ and one-third produced IL-2 after stimulation (Fig. 4b). Treg depletion led to a significant increase in the frequencies of IFN-γ^+^ cells, again, only among CD8αβ T cells in tumors (Fig. 4b). All other analyzed cell subsets displayed unchanged frequencies of IFN-γ (Fig. 4b and supplementary Fig. 9). Analysis of the combinations of different cytokines expressed by CD8αβ T cells in tumors revealed that IFN-γ^+^, IFN-γ^+^ IL-2^+^, and IFN-γ^+^ IL-2^+^ TNF^+^ cells were the most abundant populations (Fig. 4c).

In contrast to IFN-γ and IL-2, IL-17A expression was low in CD8αβ, CD8αα, and TCRγδCD8^+^ T cells, regardless of the tissue type analyzed or Treg depletion, but prominent in TCRγδCD8^−^ cells from the tumors. The inflammatory cytokine TNF could be detected only in CD8αβ and TCRγδCD8^−^ T cells, both in unaffected tissue and in tumor tissue (Fig. 4b and supplementary Fig. 9). However, Treg depletion did not affect frequencies of IL-17A- or TNF-secreting cells. Based on these results, the TCRγδCD8^−^ cells stand out as the major IL-17A producers in the tumors. When we analyzed the distribution of different combinations of cytokines produced in TCRγδCD8^−^ T cells, the frequencies of IL-17A^+^ and TNF^+^IL-17A^+^ cells were significantly higher in tumors compared to unaffected tissue (supplementary Fig. 10).

Altogether, Treg depletion has a significant effect on enhancing the frequencies of IFN-γ producing CD8αβ T cells in tumors. Furthermore, TCRγδCD8^−^ T cells secreting both IL-17A^+^ and TNF^+^ are significantly increased in tumor tissue, compared to unaffected tissue.

### CD8αβ T cells in tumors increase expression of checkpoint receptors after Treg depletion

**Fig. 5:**
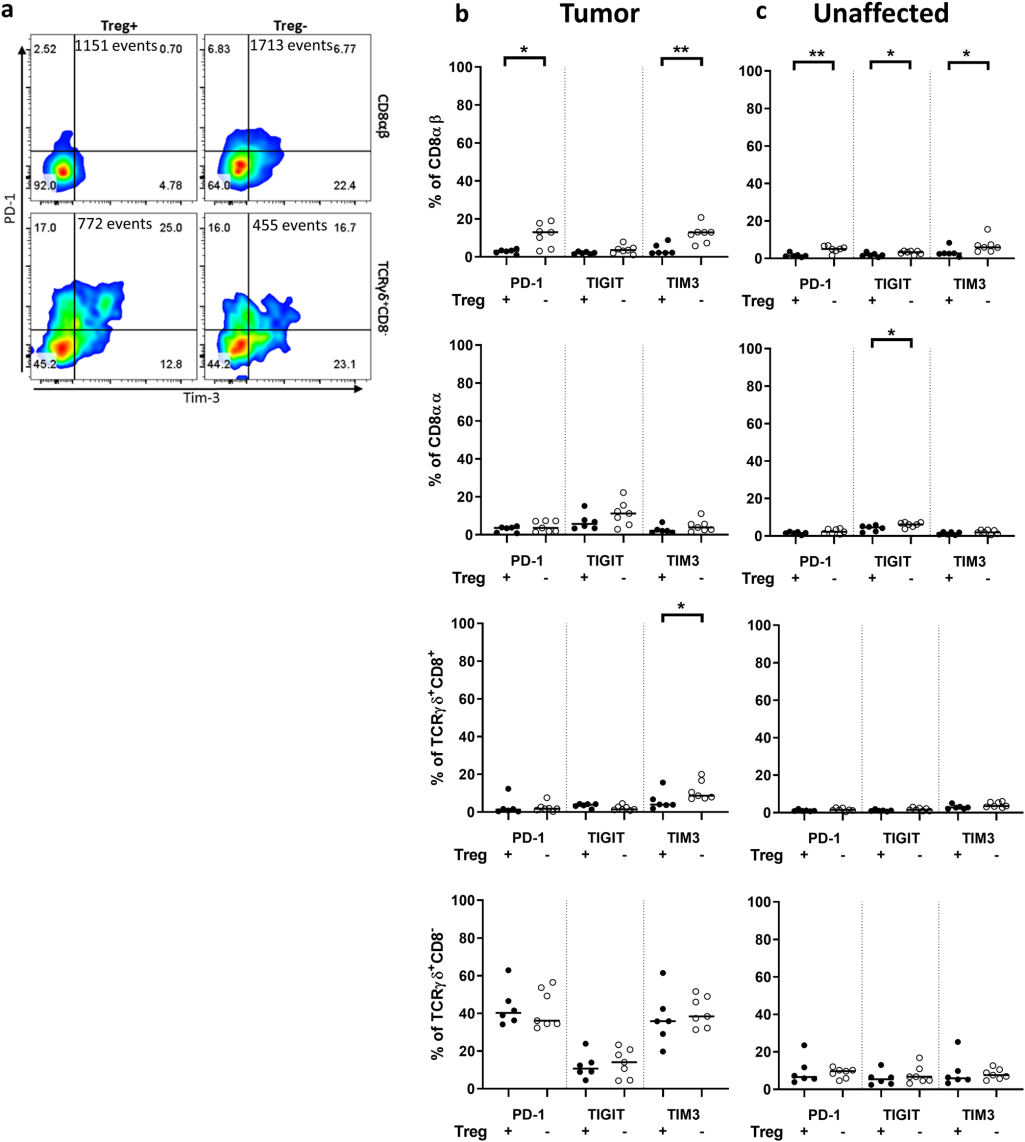
T cell expression of inhibitory receptors following Treg depletion. Single cell suspensions were isolated from intestinal tumor and unaffected tissue of DT-treated APC^Min/+^ and APC^Min/+^/DEREG mice, and analyzed by flow cytometry ex vivo. **a** Representative flow cytometry plot of CD8αβ T cells (upper panel) and TCRγδ^+^CD8^−^ T cells (lower panel) expressing PD-1 and Tim-3 in tumor tissue from Treg-proficient (Treg+) and Treg-depleted mice (Treg−). Total events are shown. Frequencies of PD-1^+^, TIGIT^+^ and Tim-3^+^ T cells in tumor tissue **(b)** and unaffected tissue **(c)** from Treg+ and Treg− mice. Symbols represent individual values and the line represents the median, *p < 0.05, **p < 0.01 using the Mann–Whitney test

We addressed the possible exhaustion of T cells by analyzing the surface expression of the commonly targeted checkpoint receptors PD-1, TIGIT, and Tim-3 (Fig. 5a). Expression of the checkpoint receptors was very low in all cell types in the control mice, except for the tumor-resident TCRγδCD8^−^ T cells, where about 40% of the cells expressed PD-1 or Tim-3. After Treg depletion, the frequencies of PD-1^+^ and Tim-3^+^ CD8αβ T cells were increased in tumors (Fig. 5b). On the other hand, depletion of Treg had little to no effect on any of the checkpoint markers on CD8αα and TCRγδCD8^+^ T cells neither in tumors (Fig. 5b) nor in unaffected tissue (Fig. 5c). The analysis of the combinations of ICOS and the checkpoint molecules showed that the majority of the PD-1^+^ and Tim-3^+^ TCRγδCD8^−^ T cells in the tumors co-expressed ICOS (data not shown), indicating that these cells may express PD-1 and Tim-3 as a result of recent activation, rather than exhaustion. In contrast, this was not observed for CD8αβ T cells.

In summary, Treg depletion increased expression of PD-1 and Tim-3 on CD8αβ T cells specifically in the tumors. PD-1 and Tim-3 expressing TCRγδCD8^−^ T cells were abundant in tumors, regardless of Treg depletion.

### PD-1 antibody treatment has no additional effect on activation and proliferation of CD8αβ T cells

**Fig. 6:**
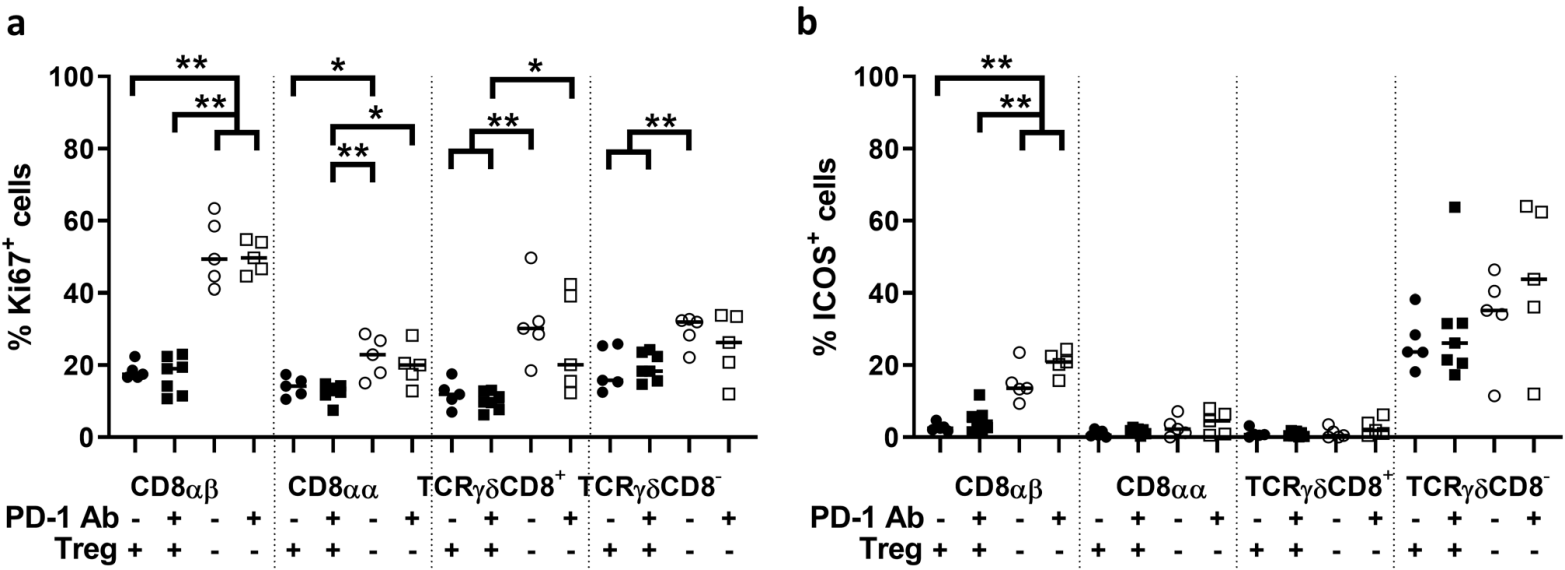
T cell expression of Ki67 and ICOS in intestinal tumors after combination of Treg depletion and blocking of PD-1. Single cell suspensions were isolated from intestinal tumors of DT-treated APC^Min/+^ and APC^Min/+^/DEREG mice and analyzed by flow cytometry ex vivo. Frequencies of T cells positive for Ki67 **(a)** and ICOS **(b)** in tumor tissue after treatment with PD-1 antibody or corresponding isotype control of Treg competent or Treg-depleted mice. From left to right: (1) isotype control with Treg, (2) anti-PD-1 antibody with Treg, (3) isotype control without Treg, (4) anti-PD-1 antibody without Treg. Symbols represent individual values, and the line represents the median, *p < 0.05, **p < 0.01 using the Mann–Whitney test

Treg in tumors express PD-L1 and PD-L2, the ligand to PD-1 [32]. We were interested to what extent Treg reduced conventional T cell activity by the PD-1–PD-L1 axis, as CD8αβ T cells in intestinal tumors increased their expression of PD-1 after Treg depletion. We treated mice with either DT or PD-1 antibody, or a combination of both, to deplete Treg and simultaneously block the interaction of PD-1 on T cells to examine whether there would be an additive effect of combined treatment. When we analyzed the proliferation of T cells by Ki-67 expression and the activation by ICOS expression in the different T cell subsets, there was no effect of the PD-1 antibody alone (Fig. 6a, b). In addition, no further increase in activation and proliferation could be detected when PD-1 blockade was added to Treg depletion, neither in tumors nor in unaffected tissue (Fig. 6a, b). Furthermore, the combination of DT and PD-1 antibody showed no additional effect on infiltration of the cell subsets into tumors nor unaffected tissue (data not shown). We conclude that PD-1–PD-L1/2 signaling alone cannot account for the effect of Treg on activation or proliferation of conventional CD8αβ T cell.

## Discussion

In this study, we used the APC^Min/+^ mouse model of spontaneous intestinal tumors to investigate the functions of different cytotoxic T cell populations in tumors compared to unaffected mucosa and the effect of Treg depletion on these populations. We could show that Treg depletion resulted in improved T cell activation, proliferation, and cytokine and GrzB production, but only of conventional TCRαβ^+^CD8αβ^+^ T cells and selectively in the tumor tissue.

We have previously shown that CD8^+^ T cells are greatly reduced in tumors of APC^Min/+^ mice [25], compared to the unaffected gut mucosa. A detailed analysis now shows that both CD8αβ and CD8αα T cells are reduced in tumors, compared to unaffected mucosa tissue. Reduced CD8^+^ cell frequencies in CRC compared to unaffected mucosa have been shown in humans as well [21]. However, the CD8αβ and CD8αα subsets are rarely separated in other studies, although it is suggested that they have different functions [33]. Generally, infiltration of conventional CD8^+^ T cells into colorectal tumors correlates with a beneficial prognosis [7], while the role of tumor-infiltrating TCRγδ T cells is less well understood [12]. The inhibitory effects of Treg on conventional T cells and consequently their tumor-promoting activity are well known, but Treg can also reduce proliferation of TCRγδ T cells from cancer patients [34]. We demonstrate here that Treg depletion leads to a specific increase in the density of CD8αβ T cells in tumors, but not CD8αα, TCRγδCD8^+^, or TCRγδCD8^−^ T cells. The increase in conventional CD8αβ T cells is partly explained by increased cell proliferation, as measured by Ki67 expression and most likely also through migration of these cells into the tumors [29].

We present here that Granzyme B expressing CD8αβ T cells increase in the tumor after Treg depletion, while no other cell subset displays increased cytotoxic activity. Cytotoxic T cells can kill tumor cells directly and their cytotoxicity is mediated through several pathways, including release of granulae containing the cytotoxic proteins Perforin and Granzyme B or the expression of Fas-L [35]. However, it is suggested that the rapid cell death pathway mediated through Perforin and Granzyme B is the major one responsible for tumor cell killing [35]. In addition to direct cytotoxic effector functions, CRC patients with a Th1-type immune profile in the tumor microenvironment show an improved disease-free survival [10]. This conclusion is also supported by the observation that mice lacking IFN-γ have more abundant and larger tumors in the intestine in a colitis model [36]. Heterozygous loss of IFN-γ also promotes adenoma progression and induced adenocarcinoma development in the APC^Min/+^ model [37]. Here, we show that Treg depletion affects tumor-infiltrating CD8αβ T cells alone and results in increased activation as measured by ICOS expression and expression of IFN-γ specifically in the tumors. This indicates that CD8αβ T cells not only accumulate in tumors upon Treg depletion, but that their cytotoxic potential and cytokine production is also enhanced, and they could thereby mediate improved tumor cell killing. These results are in accordance with a recent report [38], where the depletion of iNKT cells in APC^Min/+^ mice led to lower mRNA expression of FoxP3 (i.e., fewer Treg) in intestinal tumors, and simultaneously, IFNγ and GrB expression increased. Our data suggest that depletion of Treg in tumors could be one way to increase cytotoxicity and Th1 polarization of conventional T cells in tumors. Still, the increased cytotoxicity and Th1 immunity did not lead to tumor reduction during this short-term treatment. One explanation might be that the infiltrating cytotoxic CD8αβ T cells show little tumor-specificity and this does not change with Treg depletion. Notably, in human MSS tumors, it is a common argument that too few neo-antigens are generated to elicit an effective adaptive immune response against the tumor [7]. One solution to this problem could be to generate immune reactions toward the tumor by, e.g., radiation therapy, and simultaneously reduce Treg activity. There are several mechanisms to reduce protective immune responses in intestinal tumors. For example, a distinct population of IL-17 producing TCRγδ T cells, referred to as γδT17 cells, has been described in CRC [39]. Notably, the γδT17 cells in human CRC also produced TNF, but were not cytotoxic. Given the observations that Th17-type immunity is detrimental in CRC [10, 40], γδT17 cells may also contribute significantly to poor patient outcome [41, 42]. Here, we show that TCRγδCD8^−^ T cells are indeed a major source of IL-17A and TNF in the tumors of APC^Min/+^ mice and that they represent an adequate model of human CRC also in this aspect. Besides the detrimental effect of IL-17A, the pro-inflammatory cytokine TNF also plays a role in intestinal polyp formation in the APC^Δ468^ mouse model [43] and TNF blockade diminishes tumor development [44]. IL-17A^+^TNF^+^ TCRγδCD8^−^ T cells might thus constitute a particularly detrimental cell population in the tumor and counteract the potential positive effect of Treg depletion on CD8αβ T cells. In contrast, a report by de Vries et al. [45] proposes that human tumor-resident TCRγδ T cells expressing PD-1 may have a potential role in the anti-tumor immune response, as they show a highly activated memory phenotype and targeting these cells by PD-1 therapy could be beneficial [45]. In our mouse model, we show that TCRγδCD8^−^ T cells are indeed expressing PD-1 and Tim-3 in tumors, while TCRγδCD8^+^ T cells do not. However, treating our mice with a PD-1 antibody showed no effect on expression of activation markers and checkpoint molecules on either of the two TCRγδ T cells populations. Generally, this relatively short PD-1 antibody treatment did not affect immune responses or tumor growth in APC^Min/+^ mice. This may be caused by the pre-existing γδT17 cells response, as Th17-like responses were recently shown to limit the benefit of PD-1 antibody treatment in MSS CRC [40].

**Fig. 7:**
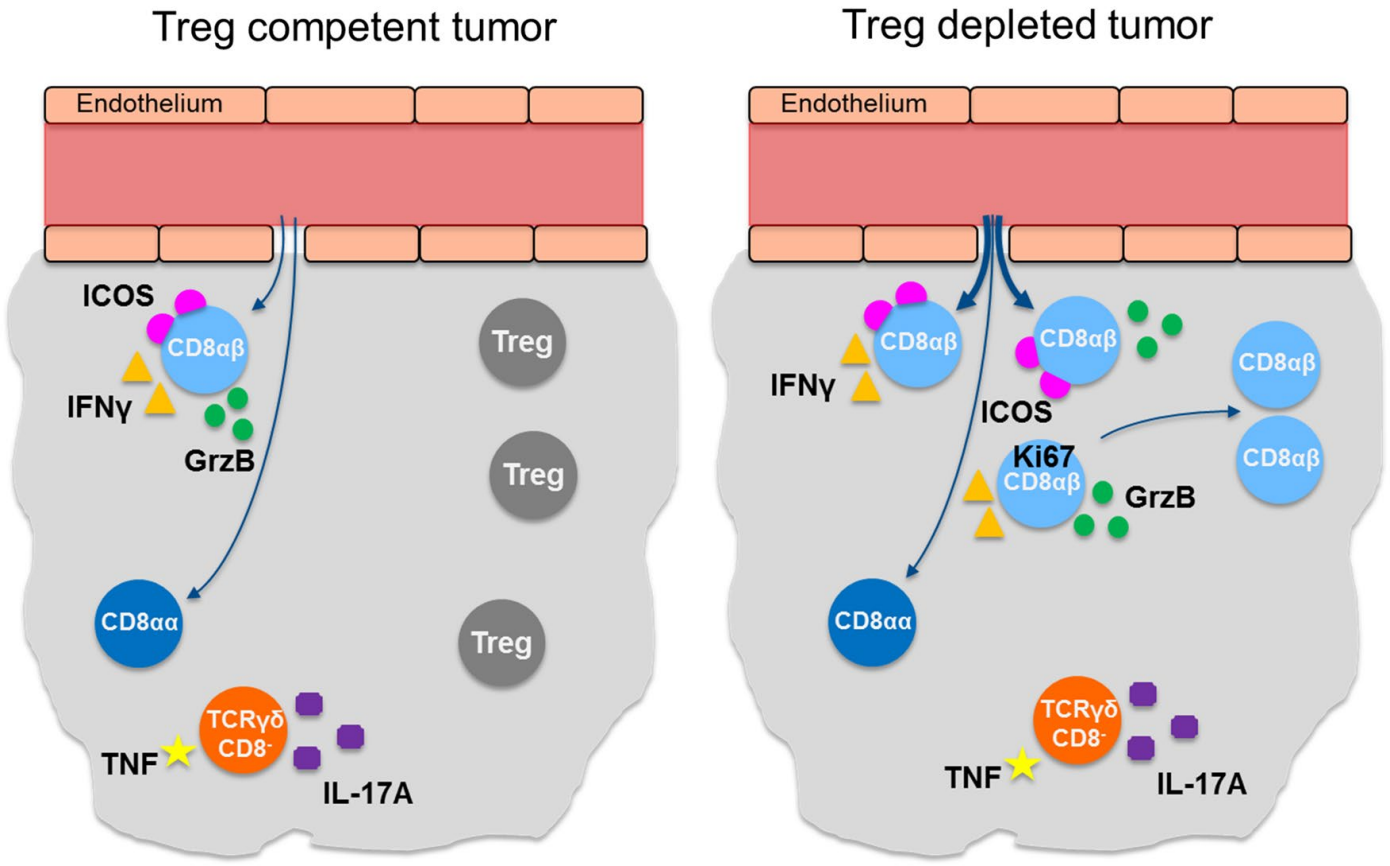
Graphical representation of the key findings in Treg competent tumors and Treg-depleted tumors. The left panel illustrates a Treg competent tumor with little migration or proliferation of CD8αβ T cells and CD8αα T cells. CD8αβ T cells express ICOS, GrzB, and IFN-γ and TCRγδ^+^CD8^−^ T cells express TNF and IL-17A. The right panel illustrates a Treg-depleted tumor where migration and proliferation of CD8αβ T cells but not of CD8αα T cells are increased. Furthermore, the frequencies of CD8αβ T cells expressing ICOS, GrzB, and IFN-γ are elevated

In summary, Treg act specifically on the subset of CD8αβ T cell, among CD8^+^ and TCRγδ^+^ T cells, in the tumor microenvironment in vivo. Depletion of Treg leads to an increase in proliferation and activation as well as expression of Granzyme B and IFN-γ in CD8αβ T cells (Fig. 7). In contrast, IL-17A and TNF secretion by TCRγδCD8^−^ T cells is not affected by Treg depletion and could counteract the CD8αβ T cell-mediated anti-tumor response. Our results show that immunotherapies aimed at depleting Treg from intestinal tumors may be a viable option for reinvigoration of conventional cytotoxic T cells with a Th1 cytokine profile. This may have to be combined with targeting of IL-17A-producing TCRγδ T cells and could lead to further improvement of infiltration and activity of cytotoxic T cells producing Th1 cytokines.

**Précis:** Regulatory T cells (Treg) suppress many types of immune cells. However, in the microenvironment of intestinal tumors, Treg suppress only conventional CD8^+^ T cells, while unconventional TCRαβ and TCRγδ T cells are not affected.

## Electronic supplementary material

Below is the link to the electronic supplementary material.

Supplementary material 1 (PDF 820 kb)

## Abbreviations

APC: Adenomatous polyposis coli
CRC: Colorectal cancer
DEREG: DEpletion of REGulatory T cell
DT: Diphtheria toxin
GrzB: Granzyme B
IEL: Intraepithelial lymphocytes
LP: Lamina propria
MSI: Microsatellite instability
MSI-H: Microsatellite instability-high
MSS: Microsatellite stable
Treg: Regulatory T cells
