# Supplementary material for: Regulatory T cells specifically suppress conventional CD8αβ T cells in intestinal tumors of APCMin/+ mice
Source: Cancer Immunol Immunother. 2020 Mar 17;69(7):1279–92. doi: 10.1007/s00262-020-02540-9 (PMC7303072; doi:10.1007/s00262-020-02540-9)
Supplement: Supplementary file 1 — Supplementary material 1 (PDF 820 kb) [file 262_2020_2540_MOESM1_ESM.pdf]

Supplementary material

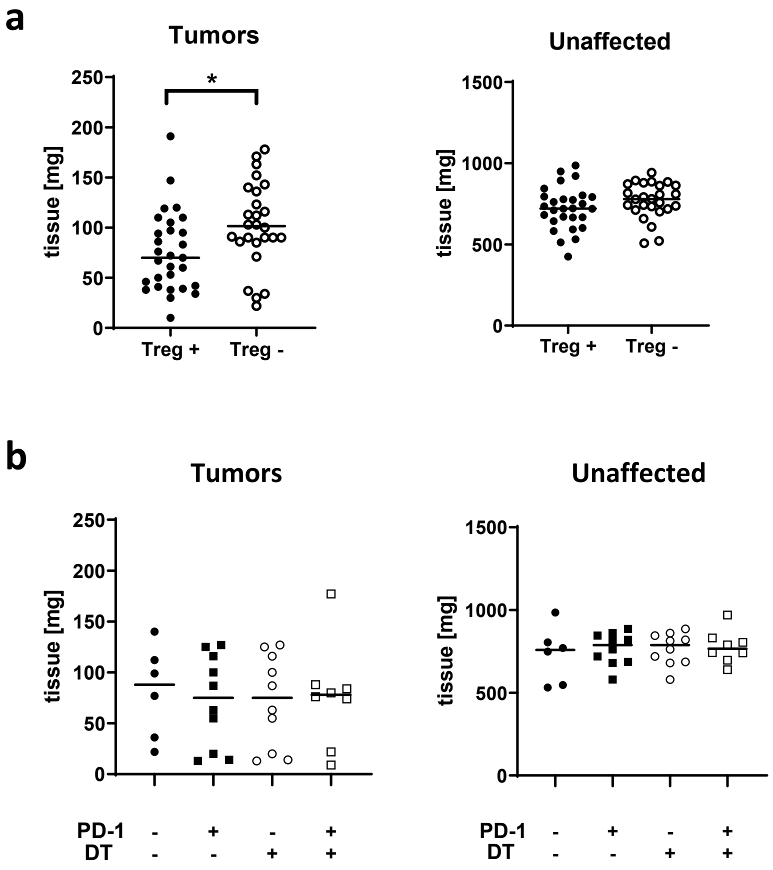

Supplementary Figure 1: Weight of all small intestinal tumors and unaffected tissue Tumor and unaffected intestinal tissue was mechanically excised from $APC^{Min/+}$ and $APC^{Min/+}$ /DEREG mice and weighed. **a)** Weight of tumors (left) and unaffected tissue (right) from Treg proficient (Treg+) and Treg depleted mice (Treg-). **b)** Weight of tumors (left) and unaffected tissue (right) from DT treated $APC^{Min/+}$ control mice, DT treated $APC^{Min/+}$ /DEREG mice and DT + PD1 antibody treated $APC^{Min/+}$ /DEREG mice. Symbols represent individual values and the line represents the median, *$p < 0.05$ using the Mann-Whitney test.

Supplementary Table 1: Antibodies with clone, dilutions and manufacturer, and Streptavidin conjugate used in flow cytometry experiments

Antibody/Streptavidin	Clone	Dilution	Manufacturer
CD45-APC/Fire™750	30-F11	1:100	Biolegend
TCRgd-PE-Cy7	GL3	1:50	Biolegend
CD4-AF700	GK1.5	1:400	Biolegend
TIGIT-PE/Dazzle™ 594	1G9	1:100	Biolegend
Tim-3-PE	RMT3-23	1:100	Biolegend
ICOS-AF647	C398.4A	1:400	Biolegend
IL-17A-AF647	TC11-18H10.1	1:200	Biolegend
TNFα-BV421	MP6-XT22	1:50	Biolegend
IFN-γ-PE	XMG1.2	1:100	Biolegend
IL-2- PE/Dazzle™ 594	JES6-5H4	1:50	Biolegend
GrzB-AF647	GB11	1:50	BD
CD8b.2-FITC	53-5.8	1:100	BD
CD8α-BUV395	53-6.7	1:100	BD
TCRbeta-BUV737	H57-597	1:50	BD
PD-1 - BV650	J43	1:100	BD
Ki67-BV421	B56	1:100	BD
Streptavidin-PE	-	1:200	Biolegend

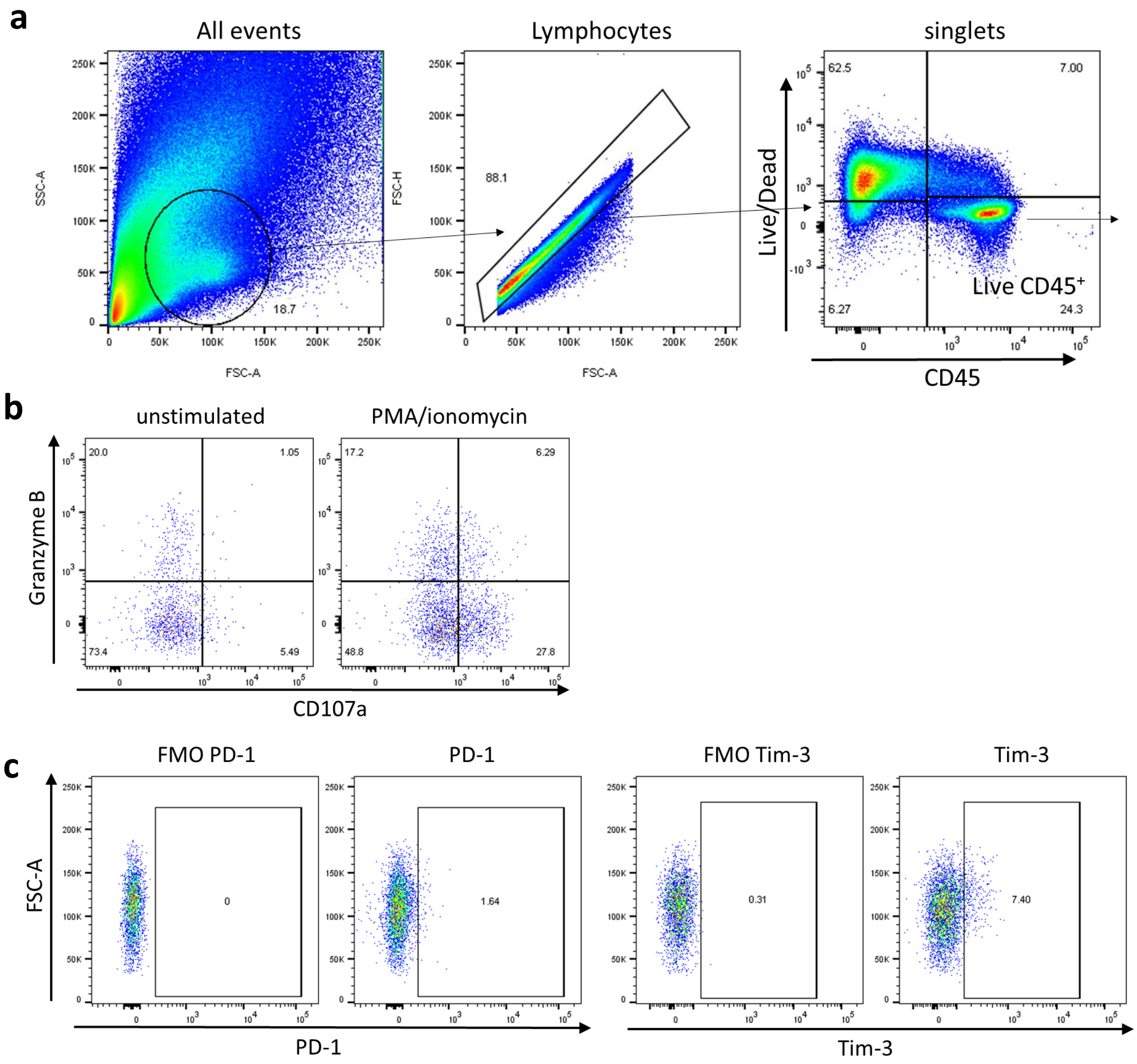

Supplementary Figure 2: Gating strategy for CD45^{+} live singlet lymphocytes and FMO controls Single cell suspensions were isolated from small intestinal tissue of APC$^{Min/+}$ mice and analysed for their expression of phenotypic markers by flow cytometry. **a)** Flow cytometry gating strategy for CD45^{+} live singlet lymphocytes. **b)** Plots show unstimulated control and PMA/ionomycin stimulation for CD107a and Granzyme B of CD8αβ T cells from unaffected small intestinal tissue. **c)** Plots show FMO controls for PD-1 and Tim-3 as well as the actual staining on CD8αβ T cells from unaffected small intestinal tissue.

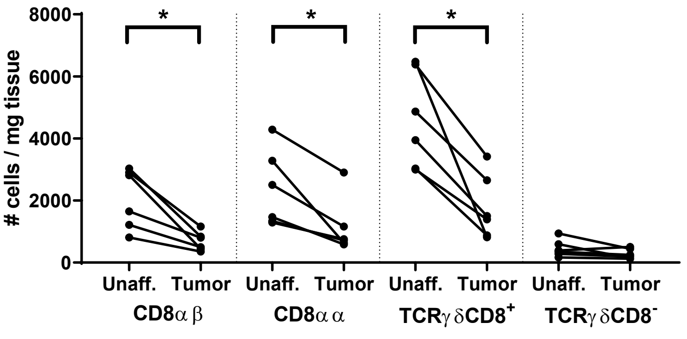

Supplementary Figure 3: T cell subsets in the intraepithelial fraction of intestinal tumor and unaffected tissue Single cell suspensions were isolated from the intrapithelial fraction of tumor and small intestinal tissue of APC$^{Min/+}$ mice and analysed by flow cytometry. Paired analysis of cell densities of the different cell populations in unaffected and tumor tissue. Symbols represent individual values, *p < 0.05 using the Wilcoxon signed rank test

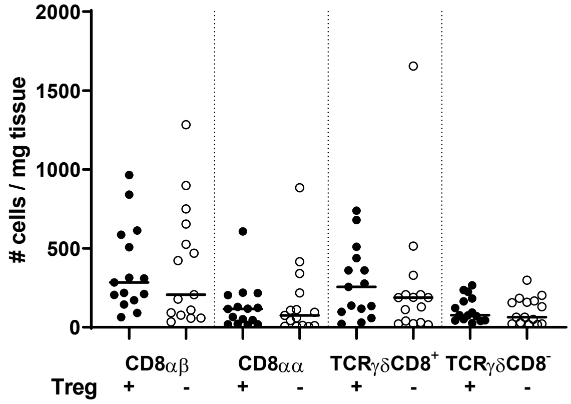

Supplementary Figure 4: T cell densities in unaffected intestinal tissue Single cell suspensions were isolated from unaffected tissue of APC$^{Min/+}$ and APC$^{Min/+}$ /DEREG mice and analysed by flow cytometry. **a)** *Ex vivo* cell densities of different T cell populations in unaffected tissue from Treg proficient (Treg+) and Treg depleted mice (Treg-). Symbols represent individual values and the line represents the median

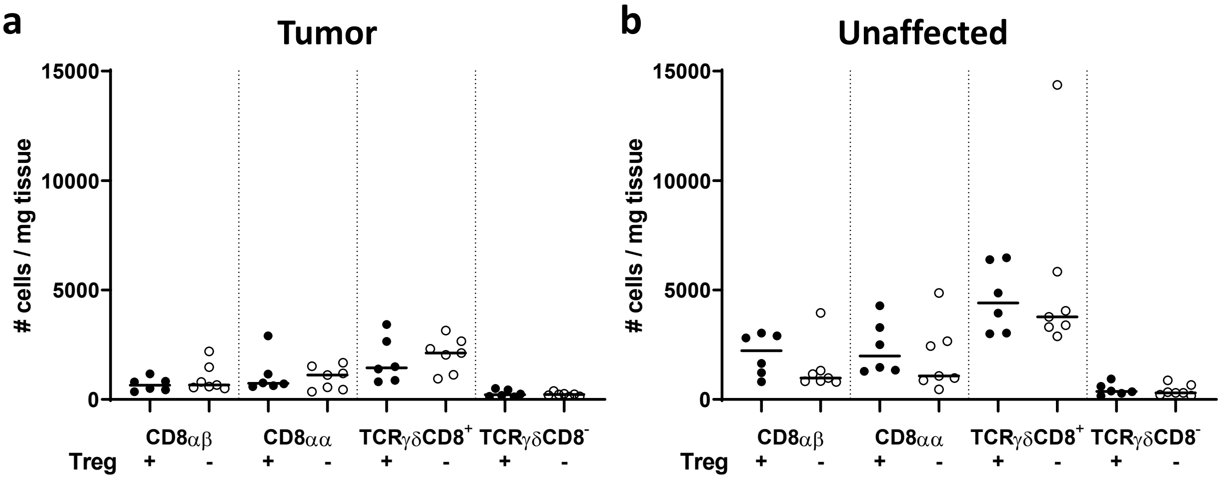

Supplementary Figure 5: T Cell densities in intraepithelial fraction of intestinal tumor and unaffected tissue Single cell suspensions were isolated from intraepithelial fractions of tumors and unaffected tissue of APC$^{Min/+}$ and APC$^{Min/+}$ /DEREG mice, and analysed by flow cytometry. *Ex vivo* cell densities of different T cell populations in tumor tissue **(a)** and unaffected tissue **(b)** from Treg proficient (Treg+) and Treg depleted mice (Treg-). Symbols represent individual values and the line represents the median

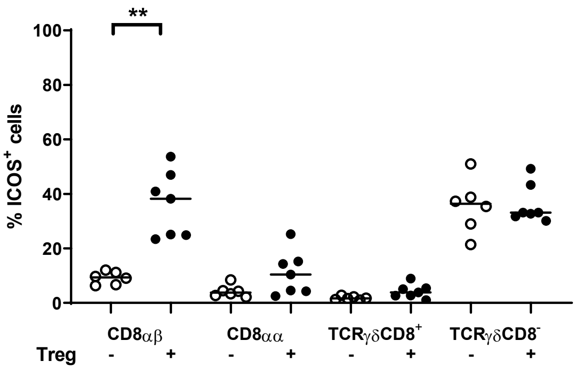

Supplementary Figure 6: T cell activation in unaffected intestinal tissue Single cell suspensions were isolated from unaffected tissue of APC$^{Min/+}$ and APC$^{Min/+}$ /DEREG mice and analysed by flow cytometry. **a)** *Ex vivo* expression of ICOS in different T cell populations in unaffected tissue from Treg proficient (Treg+) and Treg depleted mice (Treg-). Symbols represent individual values and the line represents the median, **p < 0.01 using the Mann-Whitney test

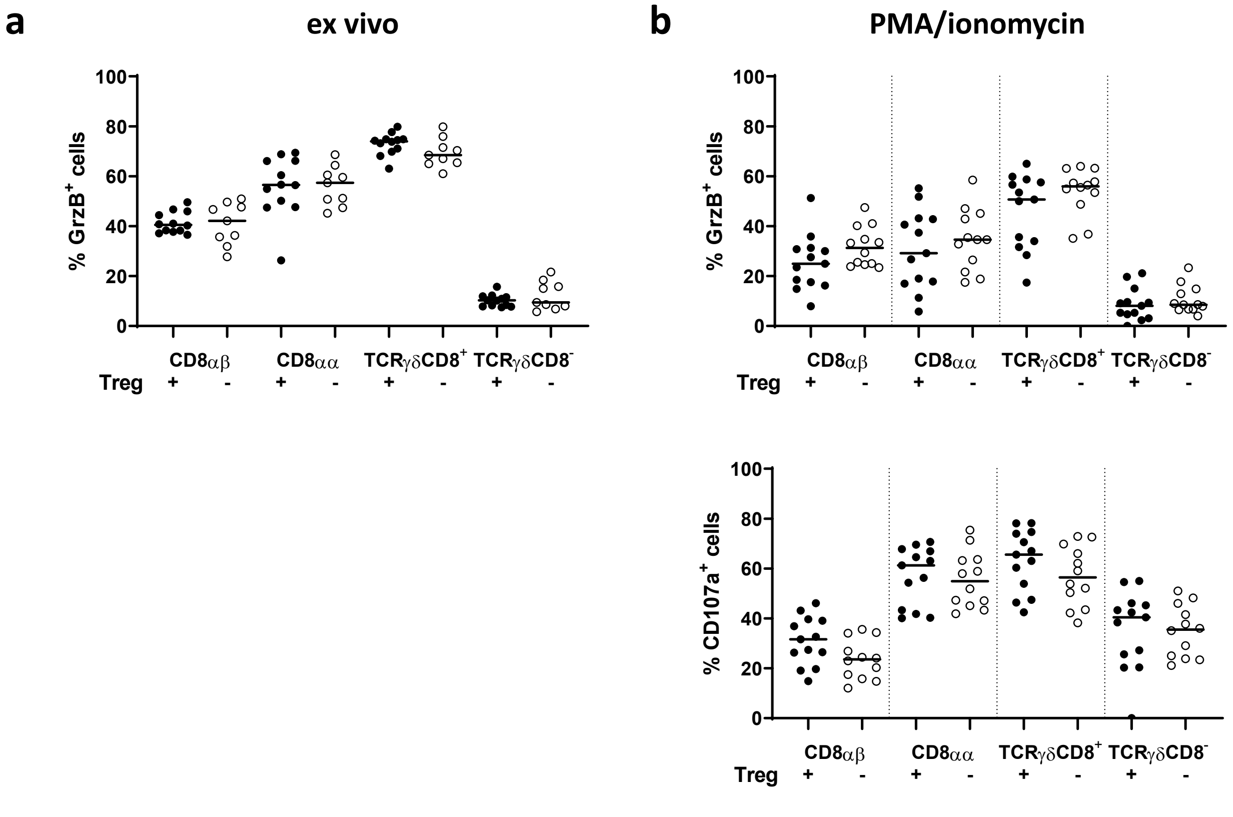

Supplementary Figure 7: T cells expressing GrzB and CD107a in unaffected intestinal tissue Single cell suspensions were isolated from unaffected tissue of APC$^{Min/+}$ and APC$^{Min/+}$ /DEREG mice and analysed by flow cytometry. Frequencies of GrzB or CD107a positive cells of different T cell populations in unaffected tissue from Treg proficient (Treg+) and Treg depleted mice (Treg-) *ex vivo* **(a)** and after stimulation with PMA/ionomycin **(b).** Symbols represent individual values and the line represents the median

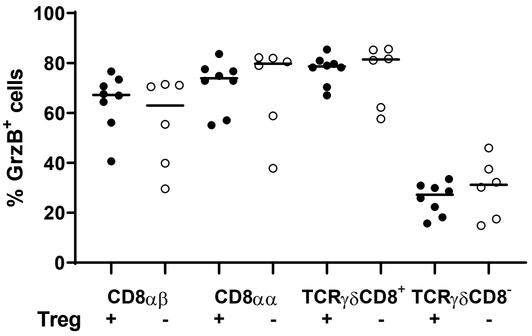

Supplementary Figure 8: T cells expressing GrzB in the intraepithelial fraction of intestinal tumors Single cell suspensions were isolated from the intraepithelial fraction of tumor tissue from APC$^{Min/+}$ and APC$^{Min/+}$ /DEREG mice, and analysed by flow cytometry. Frequencies of GrzB positive T cells *ex vivo* in tumor tissue from Treg proficient (Treg+) and Treg depleted mice (Treg-). Symbols represent individual values and the line represents the median

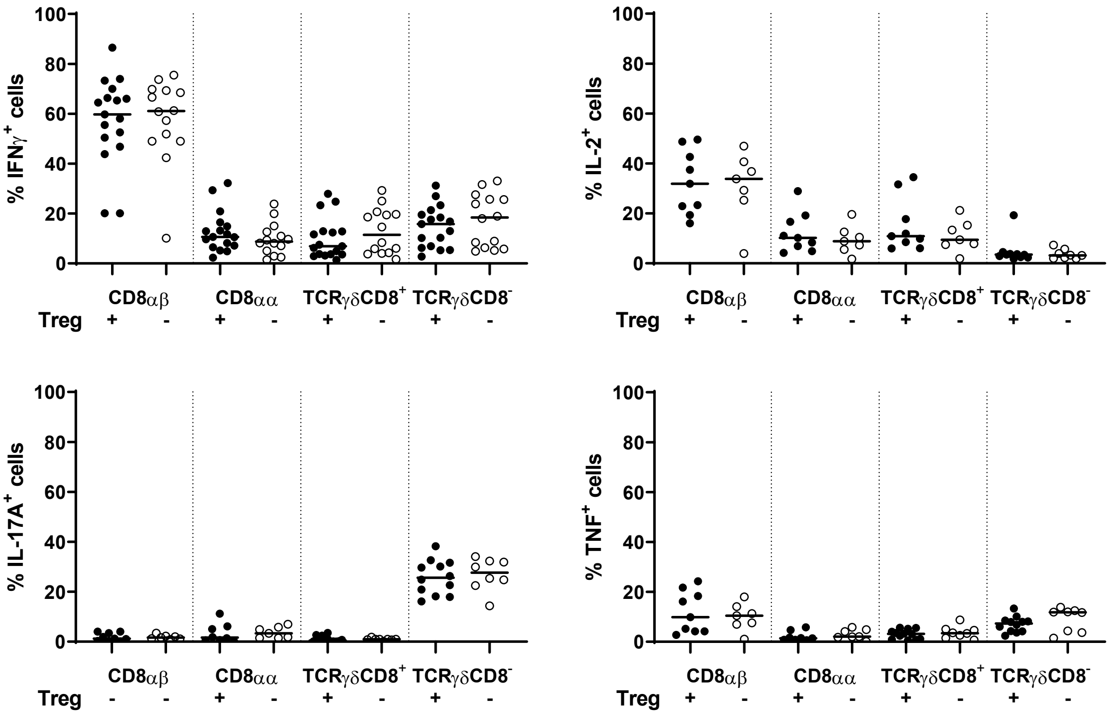

Supplementary Figure 9: Cytokine expression of T cells in unaffected intestinal tissue Single cell suspensions were isolated from intestinal tumors of APC$^{Min/+}$ and APC$^{Min/+}$ /DEREG mice and analysed by flow cytometry after PMA/ionomycin stimulation. Frequencies of IFN-γ, IL-2, IL-17A and TNF positive T cells in unaffected tissue from Treg proficient (Treg+) and Treg depleted mice (Treg-). Symbols represent individual values and the line represents the median

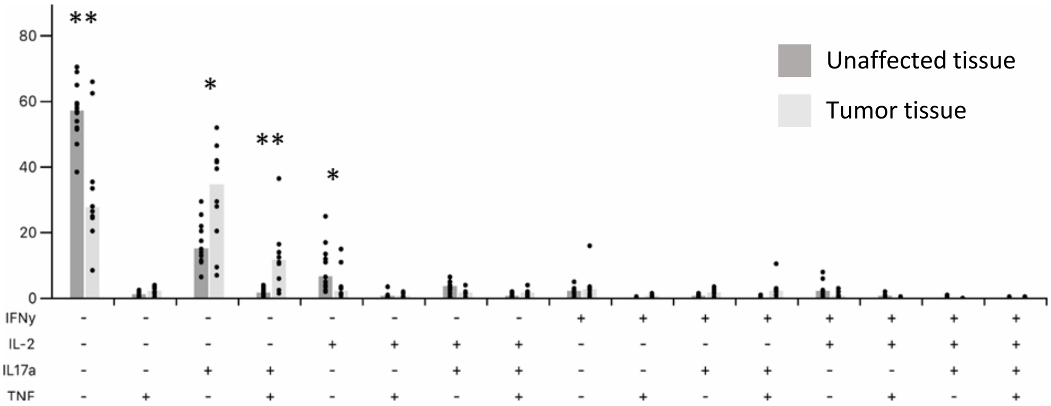

Supplementary Figure 10: Combinations of the expression of different cytokines in CD8αβ T cells from intestinal tumor tissue Single cell suspensions were isolated from intestinal tumors of $APC^{Min/+}$ and $APC^{Min/+}$ /DEREG mice and analysed by flow cytometry after PMA/ionomycin stimulation. Bar plot of all the combinations of the expression of IFN-γ, IL-2, IL-17A and TNF in TCRγδ+CD8- T cells from unaffected and tumor tissue of Treg proficient mice. Symbols represent individual values and the line/bar the median, *$p < 0.05$, **$p < 0.01$ using the Mann-Whitney test.
